# The absent/low expression of CD34 in *NPM1*-mutated AML is not related to cytoplasmic dislocation of NPM1 mutant protein

**DOI:** 10.1038/s41375-022-01593-2

**Published:** 2022-05-14

**Authors:** Giulia Pianigiani, Francesca Rocchio, Sara Peruzzi, Vibeke Andresen, Barbara Bigerna, Daniele Sorcini, Michela Capurro, Bjørn Tore Gjertsen, Paolo Sportoletti, Mauro Di Ianni, Maria Paola Martelli, Lorenzo Brunetti, Brunangelo Falini

**Affiliations:** 1 Section of Hematology, Department of Medicine and Surgery, Center for Hemato-Oncological Research (CREO), University of Perugia, Perugia, Italy; 2 Research and Early Development, Dompé Farmaceutici S.p.A, Napoli, Italy; 3 Centre for Cancer Biomarkers (CCBIO), Department of Clinical Science, University of Bergen, Bergen, Norway; 4 Department of Medicine, Haematology Section, Haukeland University Hospital, Bergen, Norway; 5 Department of Medicine and Sciences of Aging, “G. d’Annunzio” University of Chieti-Pescara, Chieti, Italy; 6 Department of Molecular and Clinical Sciences, Università Politecnica delle Marche, Ancona, Italy

**Keywords:** Acute myeloid leukaemia, Cell biology

## Introduction

*NPM1*-mutated acute myeloid leukemia (AML) represents about one-third of all adult AML [1] and, due its unique clinicopathological and genetic features [2], is recognized as a leukemia entity of the World Health Organization (WHO) Classification of myeloid neoplasms. Nucleophosmin (NPM1) is a multifunctional protein physiologically located in the nucleolus [2]. *NPM1* mutations, the most common genetic lesion in AML, abrogate the ability of the protein to localize in the nucleolus and create a new nuclear export signal (NES) at the C-terminus, leading to enhanced nuclear export of mutant NPM1 and its aberrant accumulation in the cytoplasm of leukemic cells [1, 3, 4].

We demonstrated that the interaction between mutant NPM1 and the nuclear exporter Exportin-1 (XPO1) causes the aberrant cytoplasmic delocalization of mutant NPM1 and is responsible for the high expression of HOX genes in *NPM1*-mutated AML, since relocalization of the NPM1 mutant by XPO1 inhibitors causes early downregulation of HOX genes that is followed by cell differentiation and growth arrest [2, 5]. The rapid loss of HOX expression, despite XPO1 inhibition does not restore the physiologic localization of NPM1 to the nucleolus [5], strongly suggest that the interaction between XPO1 and mutant NPM1 (rather than its localization) is responsible for maintaining high HOX levels.

Another characteristic feature of *NPM1*-mutated AML is the absent/low expression of CD34 [6, 7] that yet remains poorly investigated. This feature, combined with the low HLA-DR [8], strong CD33 expression [9] and presence of abnormal PML bodies [10], is reminiscent of acute promyelocytic leukemia (APL) and have inspired APL-like treatment strategies (i.e., all-trans retinoic acid and arsenic trioxide) also in *NPM1*-mutated AML both preclinically [10] and in patients (NCT04689815, NCT03031249). Unlike HOX genes, CD34 expression seems to be independent from XPO1-mediated cytoplasmic dislocation of mutant NPM1. This is supported by the finding that in most *NPM1*-mutated AML patients, a small subset of early CD34+ hematopoietic precursors carrying *NPM1* mutations/cytoplasmic NPM1 is usually present [11], suggesting a derivation from CD34+ hemopoietic stem cells, with the potential of multilineage differentiation. On the other hand, the observation that at least a percentage of *NPM1*-mutated AML may derive from CD34-negative hematopoietic stem cells, raises the question of a possible relationship between absent/low expression of CD34 and cytoplasmic dislocation of mutant NPM1 [12]. Clinically, CD34 expression in *NPM1*-mutated AML has been mostly associated with an adverse outcome [13].

To address this issue, we performed functional studies to assess whether the nuclear relocalization of the mutant NPM1 could result in the re-expression of CD34. Moreover, we searched for CD34+/NPM1 cytoplasmic precursors in the bone marrow (BM) biopsies of *NPM1*-AML patients at diagnosis and relapse, using a highly specific monoclonal antibody against mutant NPM1. The results of these studies are presented below.

## Results and discussion

**Fig. 1:**
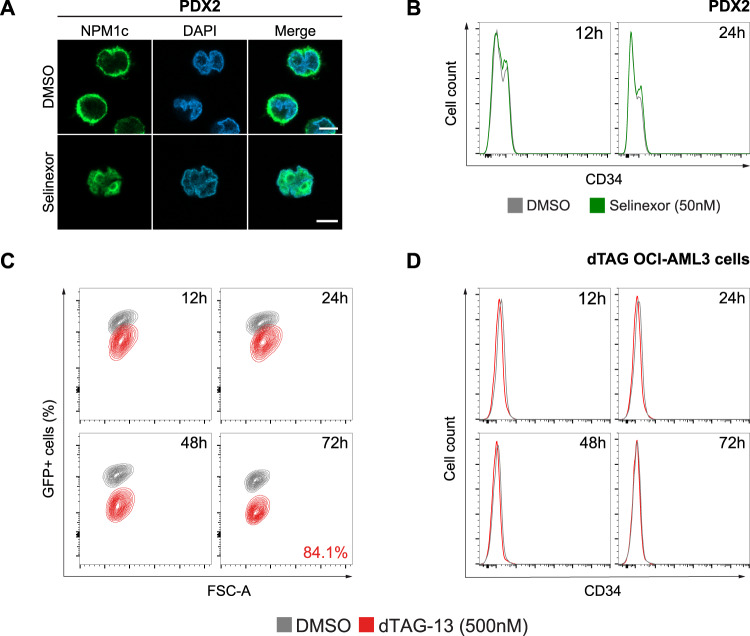
Nuclear relocalization or selective degradation of mutant NPM1 does not alter CD34 expression. **A**. Representative fluorescence microscopy images of PDX2 cells treated for 12 h with DMSO (control) or selinexor 50 nM. Cells were labeled with anti-NPM1 mutant antibody (NPM1c, green). Cell nuclei were stained with DAPI (blue). 63x magnification. Scale bar, 5 μm. **B** Representative histogram plots for CD34 expression analyzed by flow cytometry at 12 and 24 h in PDX2 cells treated with either DMSO (control, grey) or selinexor 50 nM (green). **C** Flow cytometry contour plots showing the percentage of GFP+ cells at 12, 24, 48 and 72 h in dTAG OCI-AML3 cells treated with DMSO (control, grey) or dTAG-13 500 nM (red). The degradation rate of mutant NPM1 at 72 h is shown. **D** Overlaid histogram plots representing CD34 expression analyzed by flow cytometry at 12, 24, 48 and 72 h in dTAG OCI-AML3 cells treated with either DMSO (control, grey) or dTAG-13 500 nM (red).

We first investigated whether cytoplasmic delocalization of mutant NPM1 could explain the absent/low expression of CD34 in *NPM1*-mutated AML. To this end, we assessed the effect of nuclear relocalization of mutant NPM1 on CD34 expression, using the selective XPO1 inhibitor selinexor, which blocks the nuclear export of XPO1 cargo proteins, including NPM1. We treated the *NPM1*-mutated OCI-AML3 cell line that does not express CD34 and two *NPM1*-mutated patient-derived xenograft (PDX) cells (PDX2 and PDX3) [5] that partially express CD34 with selinexor for 24 h. Immunofluorescence with a monoclonal antibody specific for mutant NPM1 showed that after 12 h of XPO1 inhibition, mutant NPM1 was completely relocated to the nucleus of AML cells (Fig. 1A, PDX2). Flow cytometry analysis performed at 12 and 24 h of treatment revealed that CD34 expression levels remained unchanged (Fig. 1B). CD34 expression was also studied by real-time PCR, confirming its low/absent expression in *NPM1*-mutated AML cells and showing that levels did not change following XPO1 inhibition (not shown).

Selinexor does not specifically block the nucleo-cytoplasmic shuttling of mutant NPM1, abrogating also the nuclear export of all XPO1 cargo proteins. Therefore, we tested the effect of selective mutant NPM1 degradation on CD34 expression. For this purpose, we exploited two *NPM1*-mutated CRISPR-engineered cell lines (OCI-AML3 and IMS-M2) in which endogenous mutant NPM1 is fused to the FKBP^F36V^ degron tag and GFP (NPM1c-FKBP^F36V^-GFP, Supplementary Fig. S1A) [5]. This system enables fast and specific degradation of mutant NPM1 by addition of the small compound dTAG-13. We treated cells with either DMSO (control) or dTAG-13 for 72 h. As expected, dTAG-13 induced efficient degradation of mutant NPM1 in both CRISPR-engineered cell lines (Fig. 1C and Supplementary Fig. S1B). Immunoblotting confirmed the loss of fusion protein without changes in wild-type NPM1 (Supplementary Figs. S1D, E). Nonetheless, flow cytometry and overlaid histograms of CD34 expression showed that CD34 levels were not changed at all time points in both cell lines (Fig. 1D and Supplementary Fig. S1C). Altogether, these findings strongly suggest that CD34 expression is not dependent upon mutant NPM1 expression and/or localization.

We then investigated the expression of mutant NPM1 in CD34+ cells of BM biopsies from *NPM1*-mutated AML patients by double immunostaining. In a previous study [11], we had addressed this issue using a monoclonal antibody recognizing both the wild-type and mutant NPM1 protein. In this study, double immunostaining for CD34/NPM1 was carried out using the highly specific anti-NPM1 mutant antibody used for immunofluorescence, suitable for automated immunoperoxidase staining of paraffin-embedded BM biopsies fixed in formalin and decalcified in Osteodec (full details are given in the Supplementary Materials).

**Fig. 2:**
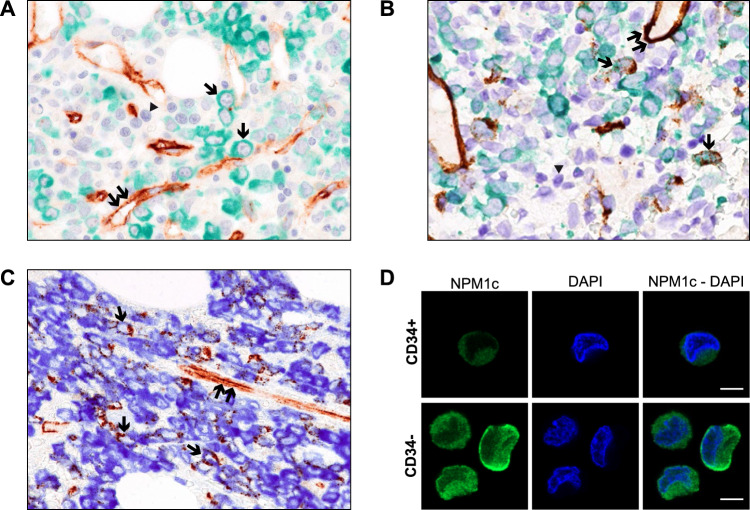
Co-expression of NPM1 mutant and CD34+ in *NPM1*-mutated AML. **A**. Formalin-fixed BM paraffin section from *NPM1*-mutated AML at diagnosis double stained for CD34 (brown, peroxidase with DAB chromogen) and mutant NPM1 protein (green, peroxidase with green chromogen, DC9913, Leica) plus BOND Polymer Refine HRP PLEX Detection and counterstaining in hematoxylin (blue). Single arrows point to leukemic cells expressing cytoplasmic NPM1 (green) but not CD34. Cells stained only in blue (hematoxylin) represent normal residual hematopoietic cells that do not express CD34 and the NPM1 mutant protein (arrowhead). Double arrows indicate CD34+ vessel endothelial cells (brown) that serve as positive control (magnification, x400). **B** Formalin-fixed BM paraffin section from another *NPM1*-mutated AML sample at diagnosis double stained with CD34 (brown) and mutant NPM1 (green) and counterstained in hematoxylin (blue) as indicated in panel A. Most leukemic cells show cytoplasmic NPM1 (green) in the absence of CD34 (brown) whilst a few blast cells (single arrows) are double stained (brown/green) for CD34 and cytoplasmic NPM1 mutant protein. Cells stained only in blue (hematoxylin) represent normal residual hematopoietic cells that do not express CD34 and the NPM1 mutant protein (arrowhead). Double arrows indicate CD34+ vessel endothelial cells (brown) that serve as positive control (magnification, x400). **C** Formalin-fixed BM paraffin section from a *NPM1*-mutated AML patient at relapse double stained for CD34 (brown, peroxidase with DAB chromogen) and mutant NPM1 protein (blue, peroxidase with blue chromogen, DC9896, Leica) plus BOND Polymer Refine Detection, without hematoxylin counterstaining. A significant percentage of leukemic cells (single arrows) are double stained (brown/blue) for CD34 and NPM1 mutant protein. Double arrows indicate CD34+ vessel endothelial cells (brown) that serve as positive control (magnification, x400). **D** Immunofluorescence with anti-NPM1 mutant antibody (green) on the CD34+ and CD34− sorted cells. Nuclei were stained with DAPI (blue). 63x magnification. Scale bar, 5 μm.

BM paraffin sections from 15 *NPM1*-mutated AML patients (*n* = 10 at first diagnosis and *n* = 5 at relapse) were double stained for CD34 and mutant NPM1. Fourteen out of the 15 cases showed the presence of no or rare CD34/mutant NPM1 double-positive cells (Fig. 2A, B). One case of *NPM1*-mutated AML at relapse (*FLT3*-ITD positive) showed a higher percentage (up to 20%) of leukemic cells double stained for CD34 and cytoplasmic mutant NPM1 (Fig. 2C). Moreover, we performed immunofluorescence using the same anti-NPM1 mutant antibody in FACS-sorted CD34+ and CD34− leukemic cells from a 32-year-old female patient with high count *NPM1*-mutated AML (Supplementary Fig. S2A). Purified CD34+ and CD34− cells were spotted onto poly-L-lysine coated glass slides and immunostained with the specific anti-NPM1 mutant monoclonal antibody followed by anti-rabbit Alexa Fluor 488-conjugated secondary antibody (full details are provided in Supplementary Materials). Both CD34+ and CD34− leukemic cells showed cytoplasm-restricted positivity for the NPM1 mutant protein without nuclear staining (Fig. 2D).

Collectively, the above results strongly suggest that, unlike HOX genes [5], CD34 expression is not dependent on the expression or the delocalization of NPM1 mutant protein from the nucleus to the cytoplasm. In fact, neither nuclear relocalization nor selective degradation of mutant NPM1 induced changes in CD34 expression in our parental and CRISPR-engineered AML cell lines.

In conclusion, based on our functional and immunohistochemical studies, we hypothesize that most *NPM1*-mutated cells originate from an immature CD34+ hematopoietic cell [10], but the leukemic bulk population is detected at an abnormal differentiation stage in which HOX expression is still high whereas CD34 is already silenced. In this context, a minor “stem-like” population, which contains leukemia-initiating cells, and which may expand at relapse [11, 14], maintains both CD34 and high HOX levels. Indeed, as in the patient with high percentage of CD34+ cells described here, an increased CD34 expression at relapse has been reported in *NPM1*-mutated AML, particularly in association with *FLT3*-ITD [11, 15].

## Supplementary information

Supplementary Information
