# Supplementary material for: The absent/low expression of CD34 in NPM1-mutated AML is not related to cytoplasmic dislocation of NPM1 mutant protein
Source: Leukemia. 2022 May 14;36(7):1931–4. doi: 10.1038/s41375-022-01593-2 (PMC9252915; doi:10.1038/s41375-022-01593-2)
Supplement: Supplementary file 1 — Supplementary Information [file 41375_2022_1593_MOESM1_ESM.pdf]

Supplementary Information

Supplementary Materials

Immortalized and primary AML cell lines

Parental OCI-AML3 cells (DSMZ, ACC 582), that harbor *NPM1* mutation A and do not express CD34 antigen, FKBP-based degron-tagged (NPM1c-FKBP^{F36V}-GFP) OCI-AML3 and IMS-M2 cells, holding NPM1 mutant allele fused to FKBP12^{F36V} and GFP, were cultured in RPMI-1640 medium supplemented with 10% Fetal Bovine Serum (FBS). 1% L-glutamine (Glu) and 1% penicillin/streptomycin (P/S) were added to cell media. All cell lines were grown in mycoplasma-free conditions and maintained in 5% CO_2 at 37°C at a concentration of 0.5x10^6 cells/ml. Xenograft (PDX) *NPM1*-mutated AML cells derived from patients after the signature of a written informed consent form, were grown in IMDM medium supplemented with 10% FBS, 1% Glu, 1% P/S and cytokines (30ng/ml FLT3L, 30ng/ml G-SCF and 15ng/ml TPO (all Cell Guidance Systems)) and maintained at a concentration of 2.0x10^6 cells/ml.

Treatment of OCI-AML3 and PDX cells with Selinexor

OCI-AML3 and PDX *NPM1*-mutated AML cells were plated at 0.5x10^6 cells/ml and treated with 50nM Selinexor or 0.3% DMSO (control) for 24h. At 12 and 24 hours, 0.2x10^6 cells were collected for flow cytometry analyses. 0.5x10^6 cells were collected in RNA lysis buffer for total RNA purification and subsequent analyses. 0.1x10^6 cells were spotted onto poly-L-lysine coated glass slides for subsequent immunofluorescence analysis.

Treatment of NPM1c-FKBP^{F36V}-GFP OCI-AML3 and IMS-M2 cells with dTAG-13

NPM1c-FKBP^{F36V}-GFP OCI-AML3 and IMS-M2 cells were plated at 0.5x10^6 cells/ml and treated with 500nM dTAG-13 (Tocris Bioscience) or 0.3% DMSO (control) for 72h. 0.2x10^6 cells were collected for flow cytometry analyses at multiple time points (12, 24, 48 and 72 hours). 0.5x10^6 cells were collected for both total RNA and protein purification and used for further analyses.

Flow cytometry

In each experiment, 0.2x10^6 cells were washed with PBS and stained for 20min at 4°C in the dark with the PE-conjugated anti-CD34 antibody (BD Biosciences) to assess CD34 expression levels. Excess antibody was removed by washing the cells with PBS. In all samples, cell debris were excluded based on FSC-A/SSC-A dot-plot and 7-Amino-

Actinomycin D (7-AAD, BD Biosciences) was used for dead-cell exclusion. Singlets were gated using the FSC-A/FSC-H dot-plot. Flow cytometry was performed with the BD FACSCanto II. Dynamics of NPM1c degradation upon dTAG-13 treatment was determined by GFP expression analysis. Flow cytometry data were analyzed with FlowJo 10.7 software (BD Biosciences) and expressed as fold change of the median fluorescence intensity (MFI) of the treated sample, relative to the control one. Graphs generation and statistical analyses were performed using Prism 8 (GraphPad).

Cell sorting of patient' cells

Leukemic cells were obtained from peripheral blood and isolated after written informed consent (Perugia Hospital) from a 32-year-old female patient with newly diagnosed *NPM1*-mutated AML. FITC-conjugated anti-CD34 and PerCP/Cy5.5-conjugated anti-CD45 antibodies (Beckman Coulter) were used for flow cytometry analysis and cell sorting. CD34+ and CD34- cells were sorted using the BD FACS Aria III cell sorter. Purity was higher than 90% in both fractions. Sorted cells were spotted onto poly-L-lysine coated glass slides and used for subsequent immunofluorescence analysis.

Immunofluorescence analysis

$0.1x10^6$ cells were spotted onto poly-L-lysine coated glass slides, fixed with 4% paraformaldehyde for 10min, permeabilized with 0.5% Triton X-100 for 10min and blocked with 0.5% BSA for 1h. Cells were labeled with an anti-NPM1 mutant rabbit monoclonal antibody (courtesy of V.A.) used as primary antibody diluted 1:100 in 0.5% BSA for 1h at RT. Anti-rabbit Alexa Fluor 488-conjugated antibody (Thermo Fisher Scientific) diluted 1:100 in 0.5% BSA was used as secondary antibody. Cell nuclei were counterstained with 4,6-diamino-2-phenylindole (DAPI) in ProLong Gold Antifade Mountant reagent (Thermo Fisher Scientific). Confocal fluorescent images were collected by a Zeiss LSM 800 confocal microscope equipped with a 63X immersion objective.

RNA isolation and qRT-PCR gene expression analysis

Total RNA was isolated according to manufacturer's instructions using the Quick-RNA Microprep Kit (Zymo Research) including in-column DNase I treatment. RNA quality control and quantification was performed using the NanoDrop spectrophotometer. RNA was reverse transcribed with the SuperScript IV First-Strand Synthesis System Kit (Invitrogen). RT-PCR was performed on a CFX96 Real-Time PCR system (Bio-Rad) using the iQ SYBR

Green Supermix (Bio-Rad) and custom primers. *GAPDH* was used as the housekeeping gene for normalization. Relative gene expression and fold change were calculated by the $2^{-\Delta\Delta Ct}$ method. All the primers are listed as follow: CD34 (FW: 5'-CACCCTGTGTCTCAACATGG-3', REV: 5'- GGGAGATGTTGCAAGGCTAG-3'); GAPDH (FW: 5'-TTTTGCGTCGCCAGCCGAG-3', REV: 5'-GGCGCCCAATACGACCAAAT-3').

Immunoblot analysis

Cell pellets were lysed with 2X Laemmli sample buffer (Bio-Rad) and boiled at 95°C for 5min. Total cell lysates and 5μl of the Precision Plus Protein Dual Color Standards (Bio-Rad) were separated by SDS-polyacrylamide gel electrophoresis (SDS-PAGE) on a 4-15% Mini-PROTEAN TGX Precast Protein Gel (Bio-Rad) and transferred onto PVDF membranes using the Trans-Blot Turbo transfer system (Bio-Rad). After 1h blocking in 5% non-fat dry milk in Tris-buffered saline containing 0.1% Tween 20 (TBS-T), membranes were probed overnight at +4°C with the following primary antibodies diluted in 5% non-fat dry milk: anti-β-tubulin (monoclonal mouse, 1:1000, T4026 Merck); anti-tNPM1 (rabbit polyclonal, 1:1000, HPA011384 Merck). Following primary antibody incubation, membranes were washed and probed with anti-mouse or anti-rabbit Horseradish Peroxidase (HRP)-conjugated secondary antibodies, were imaged using the Luminata Crescendo Western HRP Substrate (Merck Millipore) system and were visualized on a ChemiDoc MP imaging system (Bio-Rad). Images were captured on the chemiluminescence setting and analyzed using the Image Lab software.

Bone marrow samples and immunohistochemistry (IHC) analysis

Bone marrow (BM) biopsies were collected from 15 *NPM1*-mutated AML patients (n=10 at first diagnosis and n=5 at relapse) diagnosed at the Institute of Hematology and Bone Marrow Transplantation, Santa Maria della Misericordia Hospital, Perugia, Italy. Samples were fixed in formalin, decalcified in Osteodec (Bio-optica), dehydrated and then processed for paraffin embedding. Paraffin-embedded tissues were cut into 3-μm-thick sections, before dried overnight and subjected to antigen retrieval. Sections were immunostained for 15min with anti-CD34 mouse monoclonal antibody (clone QBEnd10, Leica), unmasked with the HIER 15min with ER2 protocol and stained for 30min with anti-NPM1 mutant rabbit monoclonal antibody (courtesy of V.A.) diluted 1:5 in BOND Primary Antibody Diluent (Leica) and unmasked with the HIER 25min with ER2 protocol. Sections were then processed in a BOND RX Stainer using the BOND Polymer Refine Detection system (DS9800, Leica) in

conjunction with the Blue Chromogen (DC9896, Leica) or the BOND Polymer Refine HRP PLEX Detection kit (DS9914, Leica) in conjunction with the Green Chromogen (DC9913, Leica), before mounted in Kaiser's glycerol gelatin (Merck Millipore). Immunohistochemical stained images were acquired with a U Plan FLN 40X/0.75 objective of an Olympus BX51 microscope equipped with an Olympus DP71 digital camera, using the TC Capture acquisition software.

Supplementary Figure S1

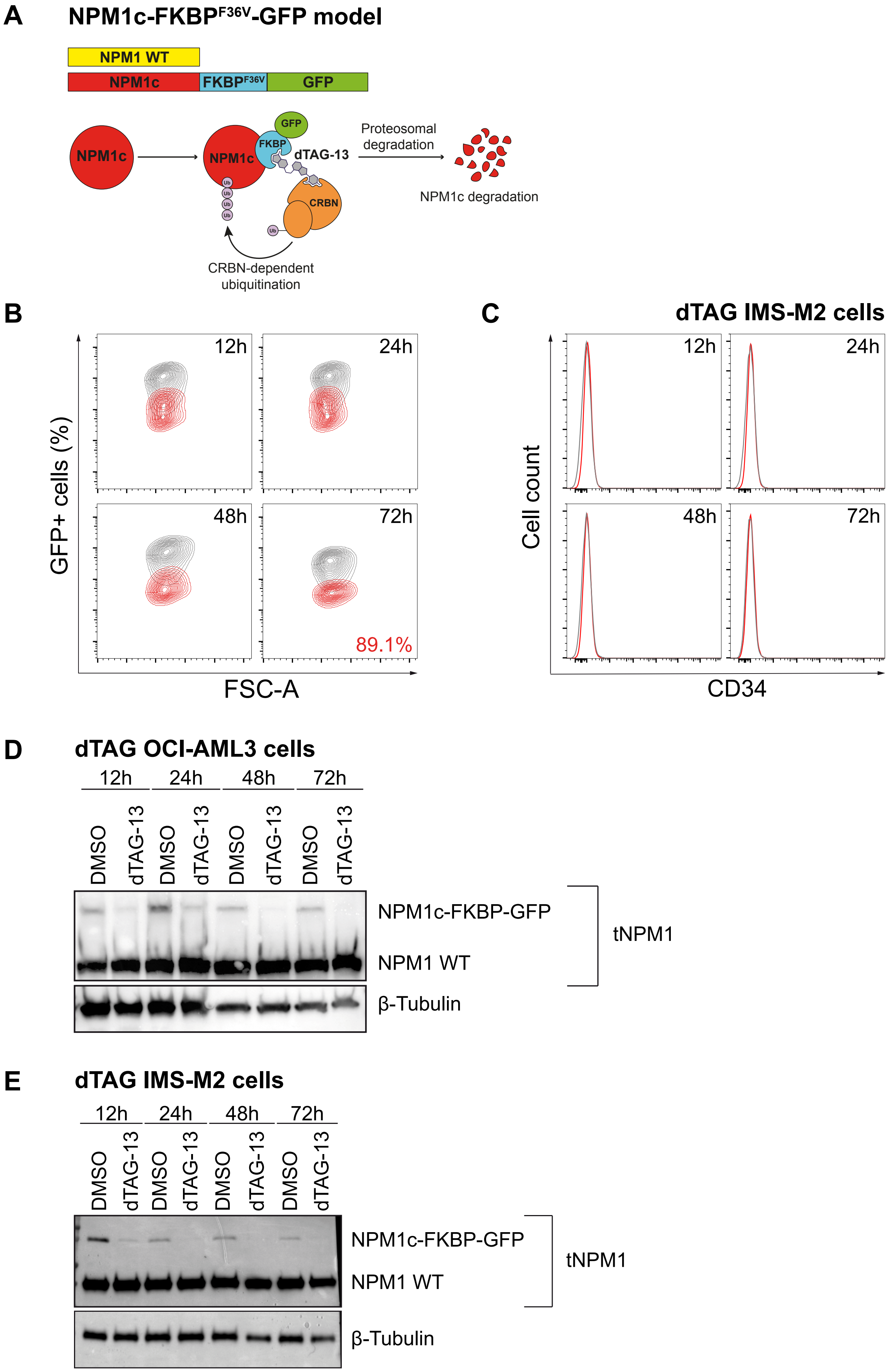

Supplementary Figure S1. dTAG-13-induced degradation of mutant NPM1.

A. Schematic illustration of degron-based technologies for selective mutant NPM1 allele degradation. Upper panel, NPM1c-FKBP^{F36V}-GFP model: CRISPR-engineered *NPM1*-mutated AML cells, holding NPM1 wild type allele (yellow box) and NPM1 mutant allele (NPM1c, red box) fused to FKBP^{F36V} (blue box) and GFP (green box). Bottom panel: the dTAG-13 degrader compound binds the FKBP-fused mutated NPM1 protein and CRL4–CRBN, causing polyubiquitination and degradation by the proteasome of the target protein. **B.** Flow cytometry contour plots showing the percentage of GFP+ cells at 12, 24, 48 and 72 hours in dTAG IMS-M2 cells treated with DMSO (control, grey) or dTAG-13 500nM (red). The degradation rate of mutant NPM1 at 72h is shown. **C.** Overlaid histogram plots representing CD34 expression analyzed by flow cytometry at 12, 24, 48 and 72 hours in dTAG IMS-M2 cells treated with either DMSO (control, grey) or dTAG-13 500nM (red). **D.** Immunoblot analysis of dTAG OCI-AML3 cells treated with DMSO (control) or dTAG-13 500nM for 12 to 72 hours. Anti-tNPM1 antibody, recognizing either wild type NPM1 and mutant NPM1 fused to FKBP^{F36V} and GFP, was used to confirm the selective degradation of mutant NPM1 in the dTAG-13 treated cells. β-Tubulin was used as loading control. **E.** Immunoblot analysis of dTAG IMS-M2 cells treated with DMSO (control) or dTAG-13 500nM for 12 to 72 hours. Anti-tNPM1 antibody, recognizing either wild type NPM1 and mutant NPM1 fused to FKBP^{F36V} and GFP, was used to confirm the selective degradation of mutant NPM1 in the dTAG-13 treated cells. β-Tubulin was used as loading control.

Supplementary Figure S2

A ***NPM1*-mutated AML patient cells**

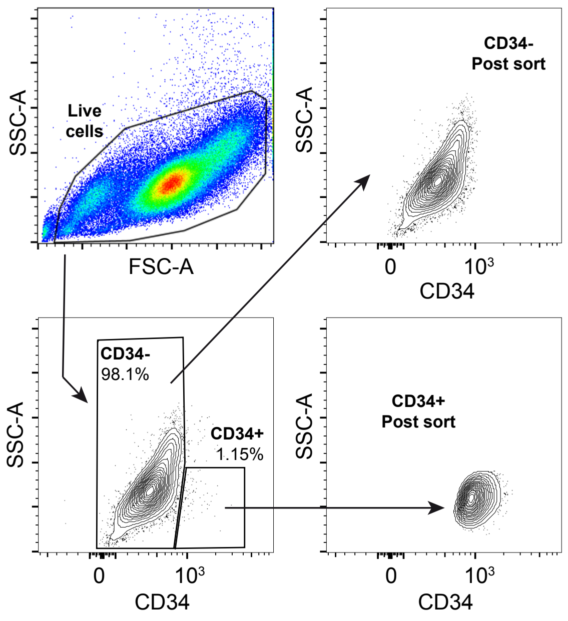

Supplementary Figure S2. Sorting of CD34- and CD34+ leukemic cells.

A. Flow cytometry analysis and cell sorting of CD34- and CD34+ leukemic cells (CD45+ population within live cells) isolated from peripheral blood of a *NPM1*-mutated AML patient at diagnosis. Sorted cells are shown in the right plots (upper, CD34- and bottom, CD34+).
